# Infective dermatitis‐like lesions as a novel skin manifestation of systemic lupus erythematosus

**DOI:** 10.1002/ccr3.2525

**Published:** 2019-12-21

**Authors:** Margot Raynal, Laure Frumholtz, Lionel Galicier, Anne Saussine, Marie‐Dominique Vignon‐Pennamen, Maxime Battistella, Martine Bagot, Michel Rybojad, Jean‐David Bouaziz

**Affiliations:** ^1^ Dermatology Department Saint‐Louis Hospital Paris France; ^2^ Immunology Department Saint‐Louis Hospital Paris France; ^3^ Université de Paris Paris France; ^4^ Pathology Department Saint‐Louis Hospital Paris France

**Keywords:** eczematous eruption, HTLV1‐associated infective dermatitis, lupus erythematosus, pustulosis

## Abstract

We describe a unique case of human T‐lymphotropic virus 1 (HTLV‐1)‐associated infective dermatitis‐like lesions in systemic lupus erythematosus. This suggests that some lupus patients may have immunological abnormalities resembling to those described in chronic HTLV‐1 infection.

## Graphical abstract

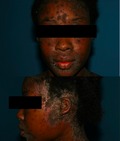

## 1 INTRODUCTION

Systemic lupus erythematosus is an autoimmune disease associated with a genetic predisposition and triggering environmental factors. Lupus skin lesions include specific skin lesions histologically characterized by an interface dermatitis (chronic, acute, and subacute lupus) and nonspecific symptoms such as alopecia, livedo, Raynaud's phenomenon, palmar erythema, periungual telangiectasia, purpura, and aseptic pustulosis of the folds. We report a case of a 17‐year‐old girl who initially presented a chronic exsudative eczematous eruption with lesions of the scalp, trunk, arm, and face with peri‐orifical distribution and frequent superinfections with staphylococcus aureus and b‐hemolytic‐streptococci. Skin lesions semiology mimicked clinical symptoms seen in Human T‐lymphotropic virus 1 (HTLV‐1)‐associated infective dermatitis (IDH). These skin lesions were associated with Evans syndrome (autoimmune hemolytic anemia and immunological thrombocytopenic purpura) and atypical colitis with a nonspecific lymphocyte infiltrate histology and anti‐Saccharomyces cerevisiae antibodies positivity. Treatment consisted of oral corticosteroid for cutaneous, hematologic, and digestive diseases that allowed almost complete remission. Nine years later, she developed systemic lupus erythematosus with skin, articular, and hematological involvement.

HTLV‐1 infection induces immune dysregulation that leads to IDH in infancy. We describe an original case of IDH‐like lesions in SLE, which suggests that some lupus patients may have immunological abnormalities resembling those described in chronic HTLV‐1 infection.

Systemic lupus erythematosus (SLE) is a chronic autoimmune disease with a pathophysiology based on the formation of self‐antibodies and immune complexes, closely related to environmental factors and genetic predispositions.^1^ Among environmental factors, viral infections can be the cause of an inflammatory trigger leading to immune dysregulation.^2^ Lupus skin lesions include erythematous edematous and squamous lesions of the face in vespertilio or diffuse lesions predominant on photo‐exposed zones, and nonspecific symptoms with alopecia, livedo, Raynaud's phenomenon, palmar erythema and periungual telangiectasia, purpura, or aseptic pustulosis of the folds.^3^ Human T‐lymphotropic virus 1 (HTLV‐1)‐associated infective dermatitis (IDH) is a chronic cutaneous disease presenting as an exsudative dermatitis. HTLV‐1 chronic infection may also be associated with several immune‐mediated disorders such as SLE.^4^

We report the case of a patient who had skin lesions clinically mimicking IDH and subsequently developed autoimmunity and SLE.

## 2 CASE REPORT

**Figure 1:**
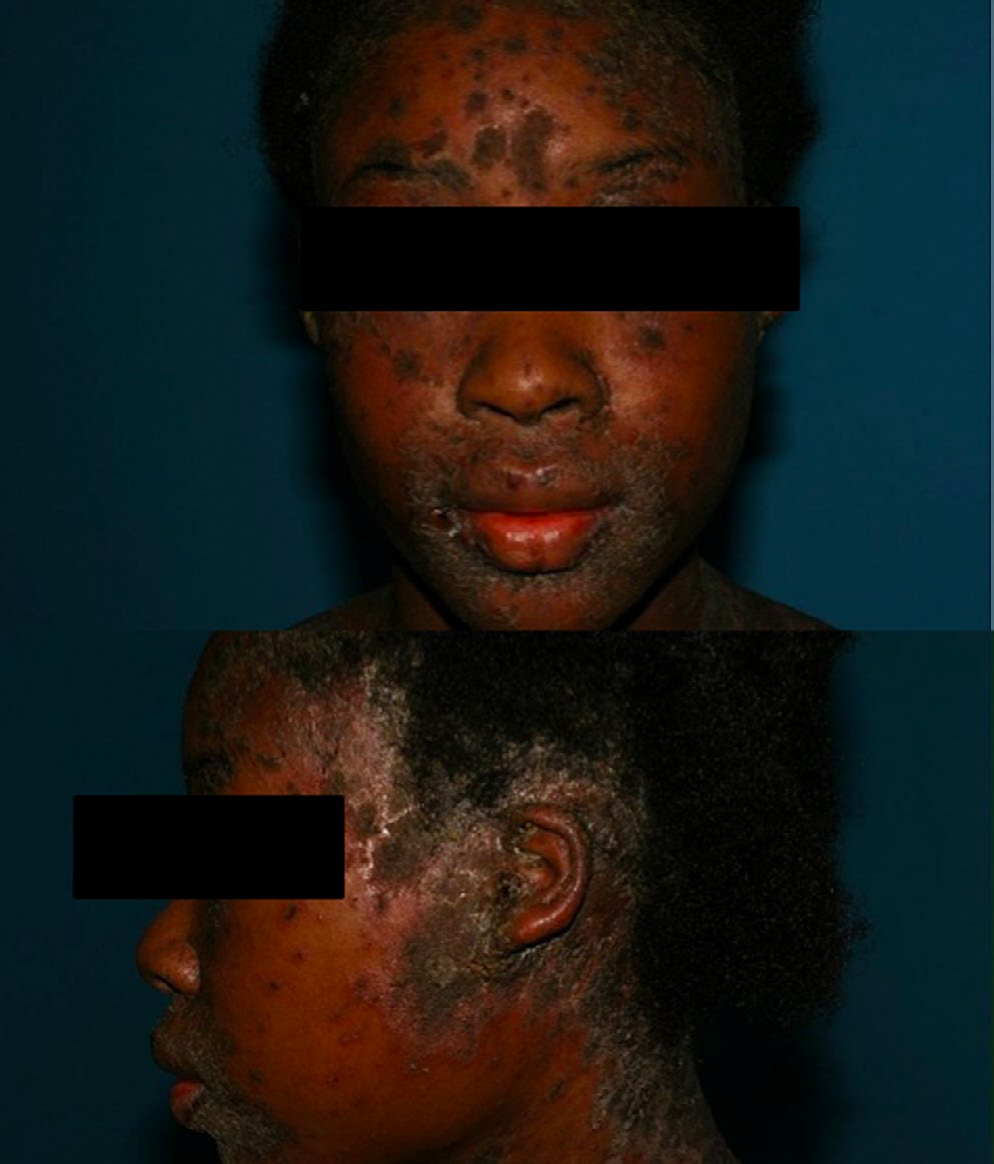
Erythematous‐scaly, exsudative, and crusted lesions of the scalp, neck and retroauricular areas, perioral, and paranasal

A 17‐year‐old girl, with Evans syndrome history (autoimmune hemolytic anemia and immunological thrombocytopenic purpura) treated with oral prednisone and IV immunoglobulins, presented with papulo‐erythematous squamous, crusted and pruriginous skin lesions of the scalp, the trunk, the back, the arms, and the face with peri‐orifical distribution (Figure 1). She also had relapsing pustules of the legs and arms*.* A trunk lesion's biopsy showed spongiotic dermatitis. A pustule's histology showed ostio‐suppurative folliculitis. Several pustule samples were positive for staphylococcus aureus and b‐hemolytic‐streptococci superinfection. Multiple antibiotic treatments improved skin lesions over short periods.

At the same time as Evans syndrome, she developed atypical colitis with a nonspecific lymphoid infiltrate on histology and anti‐Saccharomyces cerevisiae antibodies positivity. Exhaustive immune explorations researching an immune deficiency or dysregulation syndrome type LRBA deficiency were negative. HTLV1 serology and PCR were negative.

Oral corticosteroids were started for skin lesions, colitis, and Evans syndrome and drastically improved these 3 conditions, but skin lesions relapsed below 15 mg prednisone/d.

For 7 years, eczematiform and pustular rashes persisted, punctuated by short courses of corticosteroids and antibiotherapies. When she was 24 years old, she presented isolated inflammatory polyarthralgia. Biological tests showed antinuclear antidouble stranded DNA (anti‐dsDNA) antibodies and hypocomplementemia. She was diagnosed with SLE and hydroxychloroquine treatment was started (400 mg/d).

**Figure 2:**
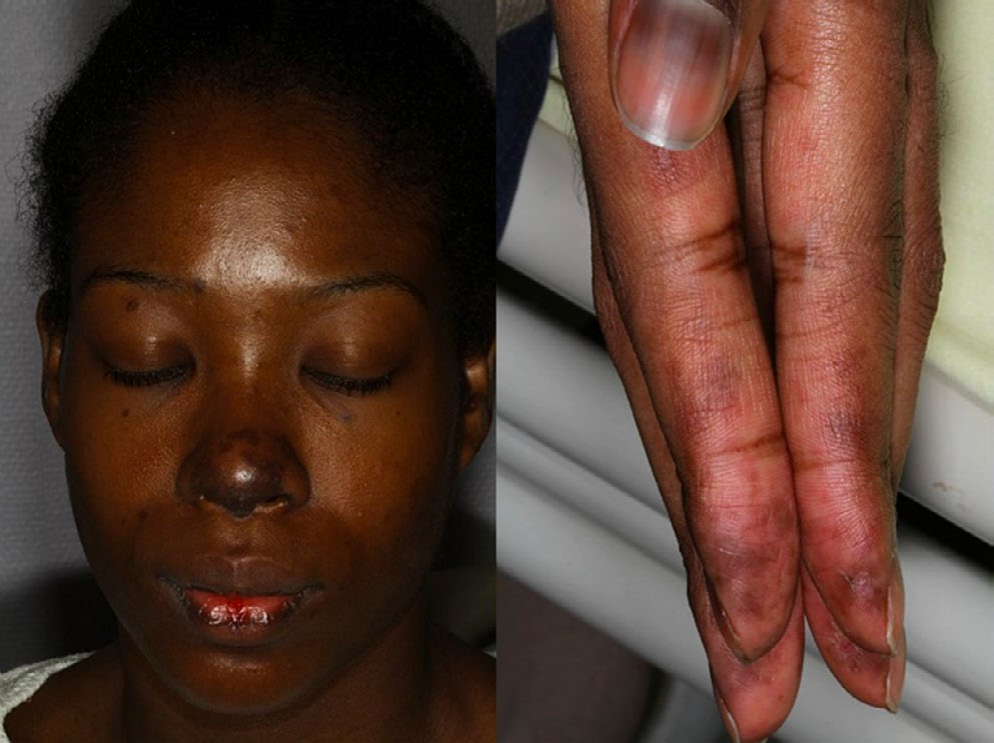
Atrophic pigmented skin lesions of the nose and ears associated with purplish‐colored fingertips papules

One year later, while she had stopped hydroxychloroquine, she had a SLE flare with hematological, cutaneous (Figure 2), and articular involvement. In addition to previous immunological abnormalities, she had anti‐Sm, RNP, ribosomal antibodies, and anticardiolipin antibodies and anti‐beta‐2‐glycoprotein‐1 antibodies. Skin biopsy of a fingertip lesion showed a vacuolar interface dermatitis consistent with acute lupus erythematosus. Combined treatment with corticosteroids (methylprednisolone dose iv 3 consecutive days, then prednisone 1 mg/kg) then oral corticosteroids and hydroxychloroquine (400 mg/d) plus low‐dose aspirin (75 mg/d) induced complete remission. Steroids were progressively tapered, and the patient remained disease‐free with 3 years follow‐up.

## 3 DISCUSSION

Thus, our patient presented two well‐distinct dermatological charts: first, an IDH‐like eruption with severe eczematous skin lesion with major facial involvement then secondarily systemic lupus erythematosus with typical cutaneous lesions.

According to infective dermatitis diagnosis criteria proposed by La Grenade et al,^5^ our patient presented 3 major criteria (erythematous‐scaly, exsudative and crusted lesions of the scalp, retroauricular areas, neck, paranasal and perioral; chronic relapsing dermatitis with prompt response to antibiotic therapy; and crusting of the anterior nares) and 3 minor criteria (positive cultures for staphylococcus aureus and b‐hemolytic‐streptococci from the skin, generalized papular rash, and generalized lymphadenopathy with dermatopathic lymphadenitis), despite HTLV‐1 negative serology. A diagnosis of amicrobial pustulosis of the fold in the setting of SLE was also discussed. However, the diffuse localization of these lesions without predominance in the folds, multiple positive bacteriological specimens, and the histology founding ostio‐suppurative folliculitis were not compatible with this diagnosis.^6^

HTLV‐1 is known to induce several diseases such as HTLV‐I‐associated myelopathy/tropical spastic paraparesis (HAM/TSP) and adult T‐cell leukemia (ATL) but may also be associated with autoimmune disorders such as Sjogren's syndrome, polyarthritis, thyroiditis, or uveitis.^7^, ^8^, ^9^, ^10^, ^11^, ^12^ The pathophysiology of these manifestations is still under investigation but immune dysregulation with lymphocyte proliferation are involved. The HTLV‐1 virus infects CD4^+^ T lymphocytes and can modify T‐lymphocyte cell function. HTLV‐1‐infected CD4^+^ T lymphocytes may exhibit altered signaling cascades and transcription factor activation, leading to changes in cell behavior that may trig inflammatory reactions that can break immune system tolerance.

Tax, an essential phosphoprotein playing a role in HTLV‐1 transcription, is known to affect several transcription factors including CREB/ATF, NF‐κB, AP‐1, SRF, and Nuclear factor of activated T cells (NFAT), as well as a number of signaling cascades involving Rho‐GTPases and Janus kinase (JAK)/signal transducer and activator of transcription (STAT), thus altering the transforming growth factor‐β (TGF‐β) cascades.^13^, ^14^ These factors are involved in cell proliferation and activation, including expression of cytokines and activation of viral proteins.

The expression of forkhead/winged‐helix transcription factor (FOXP3), which is an important transcription factor, has also been reported to be altered in patients infected with HTLV‐1. FOXP3 is an essential transcription factor for the differentiation, function, and homeostasis of regulatory T cells (Tregs). Irregularities in the expression of FOXP3 may lead to loss of immune tolerance and the probable development of autoimmune diseases*.* 4, 15, 16

Furthermore, SLE is an autoimmune disease with autoantibody formation and cell immunity disturbance. We currently find altered suppressor T‐cell to helper T‐cell ratios. Abnormalities in T‐cell function include T‐cell lymphopenia, impaired apoptosis, hyper‐reaction to signaling to T‐cell receptors, expression of activated antigens, defects in deletion of cells with high affinity for self‐antigens, and alteration of responses to cytokines and lymphokines.^17^

Thus, a possible association between SLE and HTLV‐1 has been discussed.^17^ One possible mechanism proposed for this association is a process of molecular mimicry through the endogenous sequence related to HTLV‐1 (HRES‐1) in the development of SLE. This could trigger the production of self‐antibodies, leading to the formation of immunocomplexes that are deposited in the tissues.^4^

In summary, we describe a unique case of IDH‐like lesions in SLE. This suggests that some lupus patients may have immunological abnormalities resembling to those described in chronic HTLV‐1 infection.

## CONFLICT OF INTEREST

None.

## AUTHOR CONTRIBUTIONS

First author: written the manuscript. Second author: edited and proofread the manuscript. Third and fourth authors: examined and treated the patient, analyzed and described anatomo‐pathological samples. Fifth and sixth authors: examined the patient and participated in therapeutic decisions. Seventh author: examined and followed the patient, edited and proofread the manuscript.
